# Myocardial GRK2 Reduces Fatty Acid Metabolism and β-Adrenergic Receptor-Mediated Mitochondrial Responses

**DOI:** 10.3390/ijms23052777

**Published:** 2022-03-03

**Authors:** Ruxu Zhai, Erika L. Varner, Ajay Rao, Sunil Karhadkar, Antonio Di Carlo, Nathaniel W. Snyder, Priscila Y. Sato

**Affiliations:** 1 Department of Pharmacology and Physiology, Drexel University College of Medicine, Philadelphia, PA 19102, USA; rz336@drexel.edu; 2 Center for Metabolic Disease Research, Department of Cardiovascular Sciences, Lewis Katz School of Medicine at Temple University, Philadelphia, PA 19140, USA; erikalvarner@gmail.com (E.L.V.); ajay.rao@temple.edu (A.R.); natewsnyder@temple.edu (N.W.S.); 3 Section of Endocrinology, Diabetes and Metabolism, Lewis Katz School of Medicine at Temple University, Philadelphia, PA 19140, USA; 4 Department of Surgery, Lewis Katz School of Medicine at Temple University, Philadelphia, PA 19140, USA; sunil.karhadkar@tuhs.temple.edu (S.K.); antonio.dicarlo@tuhs.temple.edu (A.D.C.)

**Keywords:** beta-adrenergic receptors, GRK2, metabolism, cardiomyocytes, mitochondria

## Abstract

G-protein coupled receptor (GPCR) kinase 2 (GRK2) is upregulated in heart failure (HF) patients and mouse models of cardiac disease. GRK2 is a regulator of β-adrenergic receptors (βARs), a GPCR involved in ionotropic and chronotropic responses. We and others have recently reported GRK2 to be localized in the mitochondria, although its function in the mitochondria and/or metabolism remain not clearly defined. We hypothesized that upregulation of GRK2 reduced mitochondrial respiratory function and responses to βAR activation. Utilizing isolated mouse primary adult cardiomyocytes (ACMs), we investigated the role of glucose, palmitate, ketone bodies, and BCAAs in mediating cell survival. Our results showed that myocyte upregulation of GRK2 promotes palmitate-induced cell death. Isotopologue labeling and mass spectrometry showed that the upregulation of GRK2 reduces β-hydroxybutyryl CoA generation. Next, using isoproterenol (ISO), a non-selective βAR-agonist, we determined mitochondrial function in mouse and human primary ACMs. Upregulation of GRK2 impaired ISO-mediated mitochondrial functional responses, which we propose is important for metabolic adaptations in pathological conditions. Increased cardiac levels of GRK2 reduced fatty acid-specific catabolic pathways and impaired ISO-stimulated mitochondrial function. Our data support the notion that GRK2 participates in bioenergetic remodeling and may be an important avenue for the development of novel pharmacological strategies in HF.

## 1. Introduction

Heart failure (HF) is the leading cause of death in the US resulting in hospitalizations, pharmacological interventions, and lifestyle alterations [1]. The β-adrenergic receptor (βAR) is a major cardiac G-protein coupled receptor (GPCR) system involved in ionotropic and chronotropic responses [1]. Increased contractile demand requires maximal efficiency in ATP-driven responses [2]. ATP is primarily derived from the oxidative phosphorylation of ADP in the mitochondrial respiratory chain [2]. Murine and human studies have shown that cardiac outcomes in HF are inversely proportional to myocardial levels of GPCR kinase 2 (GRK2) [3,4], a major regulator of cardiac βAR signaling [1]. Recently, we and others have shown that GRK2 is also localized to mitochondria [5,6,7], where the function of this enzyme in this organelle is dependent on localization and kinase activity [8]. Moreover, we and others have shown that GRK2 plays a role in metabolism, particularly in cardiomyocyte fatty acid utilization [8,9], yet specific mechanisms of action and cardiac functional outcomes remain poorly understood. Studies spanning over decades have shown that cardiac metabolism and substrate preference shifts throughout development and in pathological conditions [10,11]. Further untangling these signaling alterations is paramount to understanding the metabolic impact of GRK2 in the development of HF. In particular, it remains unclear whether GRK2 upregulation in ACMs impairs βAR-mediated mitochondrial responses. Here, we provide evidence that upregulation of cardiac GRK2, even in the absence of cardiac injury/stress, modifies bioenergetic homeostasis of ACMs, which may contribute to HF disease progression.

Utilizing a mouse model of cardiac-specific Grk2 overexpression (Grk2TG) [12], we investigated whether upregulation of GRK2 in ACMs altered metabolic pathways that may pre-dispose the heart for detrimental outcomes. Moreover, we hypothesized that upregulation of GRK2 in ACMs negatively impacts mitochondrial metabolic adaptations resulting from βAR stimulation that ultimately contribute to declined mechanical responses. These data support the notion that the mechanistic involvement of GRK2 in HF goes beyond GPCR- regulation and may provide a novel avenue for pharmacological intervention.

## 2. Results

### 2.1. Palmitate Promotes GRK2-Mediated Cell Death

**Figure 1:**
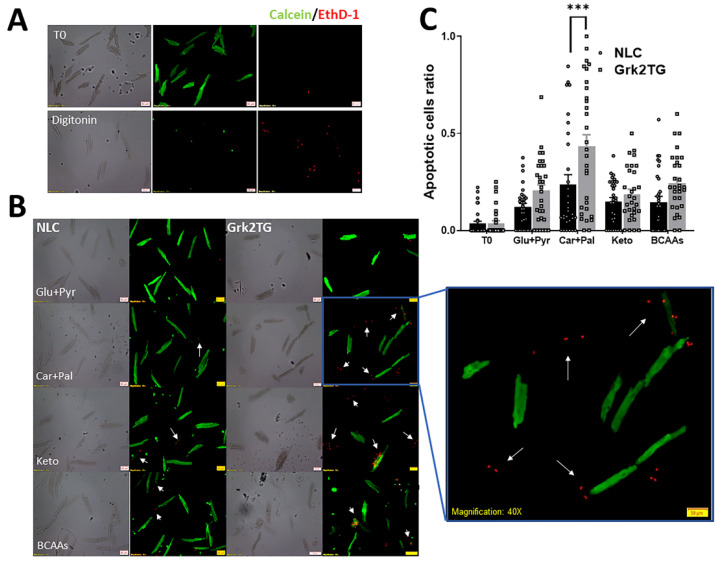
Upregulation of GRK2 in the myocyte enhances palmitate-driven cell death. (**A**) Digitonin treatment in ACMs induced cell death and labeling for EthD-1. Calcein-AM (green) labeled live cells. Scale bar is 50 µm. (**B**) Representative confocal images using calcein-AM (green, live cells) and ethidium homodimer-1 (red, dead cells) in ACMs in the presence of specific substrates. (Scale bar is 50 μm). (**C**) Quantification of B displayed as mean ± SEM. (Glucose: 0.205 0.031 Grk2TG; 0.120 ± 0.019 NLC; palmitate/carnitine: 0.433 ± 0.060 Grk2TG; 0.237 ± 0.050 NLC; ketone: 0.201 ± 0.029 Grk2TG; 0.178 ± 0.030 NLC; BCAAs: 0.244 ± 0.027 Grk2TG; 0.146 ± 0.029 NLC; *n* = 10 images/condition in 3 hearts/genotype; *** *p* < 0.0001).

Our initial experiments investigated whether GRK2 altered ACM substrate utilization and substrate-specific cell survival. ACMs from Grk2TG and non-littermate control (NLC) mice were isolated [8], cultured, and initially exposed or not to digitonin to validate labeling of dying ACMs (Figure 1A). ACMs from Grk2TG and NLC mice were then restricted to utilize glucose, palmitate, ketone bodies, or branched-chain amino acids to assess substrate-specific cell survival (Figure 1B). Live/dead experiments showed an accelerated cell death of approximately 2-fold in Grk2TG when compared to NLC ACMs cultured in palmitate (Figure 1B) suggesting an impairment in fatty acid metabolism in Grk2TG ACMs. Data quantification is shown in Figure 1C where the upregulation of GRK2 promotes palmitate-induced cell death. Time zero (T0) is the apoptotic ratio at time of ACM isolation.

### 2.2. Upregulation of GRK2 in Adult Cardiomyocytes Reduces Palmitate Catabolism

**Figure 2:**
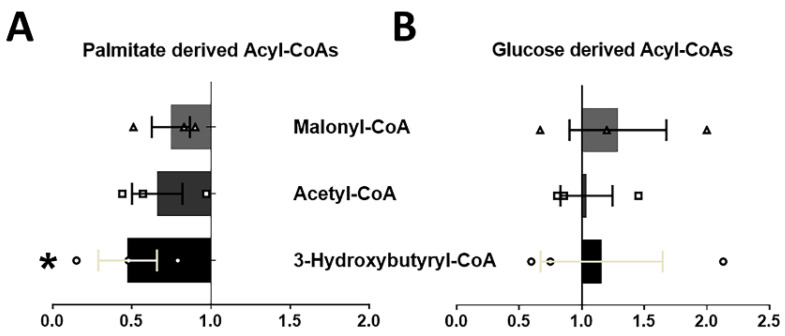
GRK2 negatively impact fatty-acid catabolism. (**A**) Palmitate-derived isotopically labeled malonyl-CoA, acetyl-CoA, and 3-Hydroxybutyryl-CoA in Grk2TG ACMs normalized to NLC. (**B**) Same as A but glucose-derived acyl-CoAs. Data were normalized to NLC (*n* = 3 hearts/condition, mean ± SEM; * *p* < 0.05).

Next, we performed fatty acid metabolic isotope tracing experiments, culturing ACMs in either ^13^C_6_-glucose or ^13^C_16_-palmitate to determine the fate of the substrates in these cells (Figure 2). We observed a decrease in palmitate-derived Acyl-CoAs, including 3-hydroxybutyryl (3HB)-CoA (Figure 2A), a major metabolite of the β-oxidation pathway, in Grk2TG ACMs when compared to NLC cells in the same conditions. This suggested a decrease in β-oxidation that is linked to a trend towards a decrease in total ^13^C-labeled Acetyl-CoA (Figure 2A). No statistical alterations were observed in isotopologues derived from ^13^C-glucose (Figure 2B); suggesting that in these cellular conditions, increases in GRK2 mainly reduce fatty acid utilization and catabolism.

### 2.3. Chronic βAR Stimulation and GRK2 Levels in Cellular Compartments Is Increased in Grk2TG Hearts

**Figure 3:**
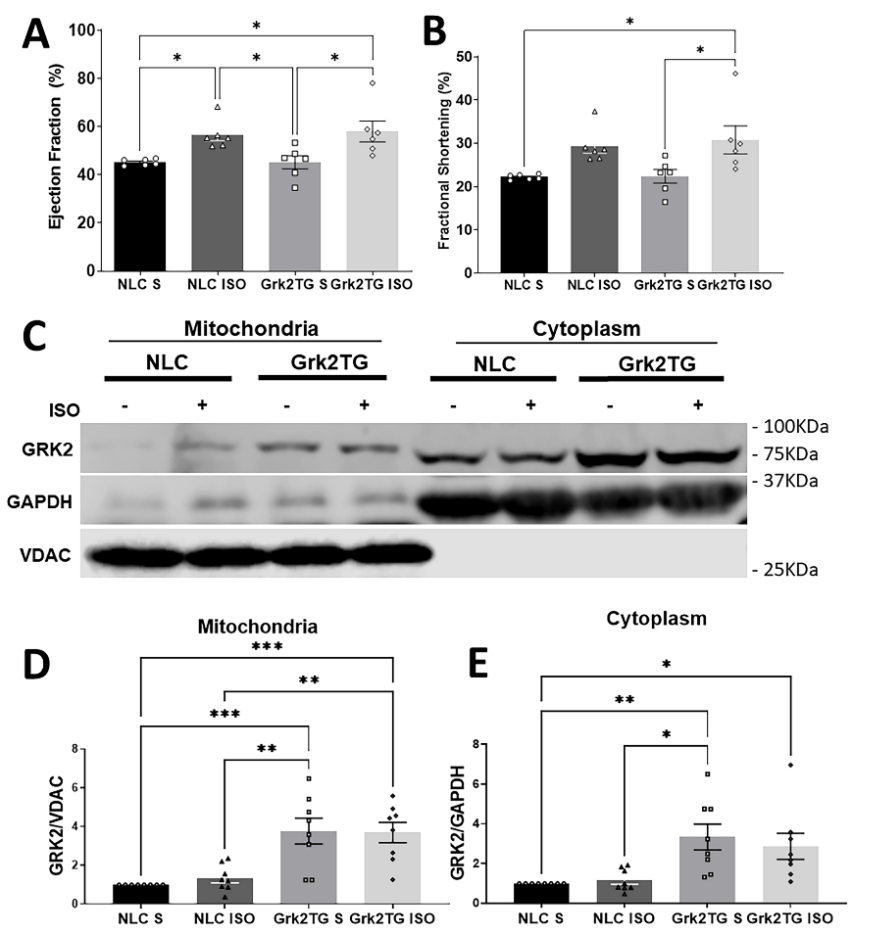
Chronic βAR stimulation and GRK2 levels in cellular compartments is altered in Grk2TG hearts (**A**) Left ventricular ejection fraction and (**B**) fractional shortening were measured by echocardiography (*n*= 6 independent mice/group). (**C**) Representative immunoblots of GRK2 expression level in mitochondria (left) and cytoplasm (right). (**D**) Mitochondrial and (**E**) cytosolic GRK2 protein quantification (*n*= 8 independent mice/group; mean ± SEM; * *p* < 0.05, ** *p* < 0.01, *** *p* < 0.005).

To investigate how chronic βAR stimulation alters ACM mitochondrial function, Grk2TG and NLC mice received isoproterenol (ISO; a non-selective βAR agonist) or saline (S) for 7 days prior to experiments. Echocardiography was performed in these mice showing an increase in ejection fraction and fractional shortening elicited by ISO stimulation (Figure 3A,B). Mitochondrial GRK2 (mitoGRK2) is increased in Grk2TG hearts when compared to NLC with an increased trend in mitoGRK2 in NLC-ISO compared to NLC S (Figure 3C–E). Cytoplasmic cardiac GRK2 levels were increased in Grk2TG mice when compared to NLC (Figure 3C–E). VDAC and GAPDH were used as loading controls.

### 2.4. Mitochondrial Functional Responses Are Altered in Response to βAR Stimulation and GRK2 Expression

**Figure 4:**
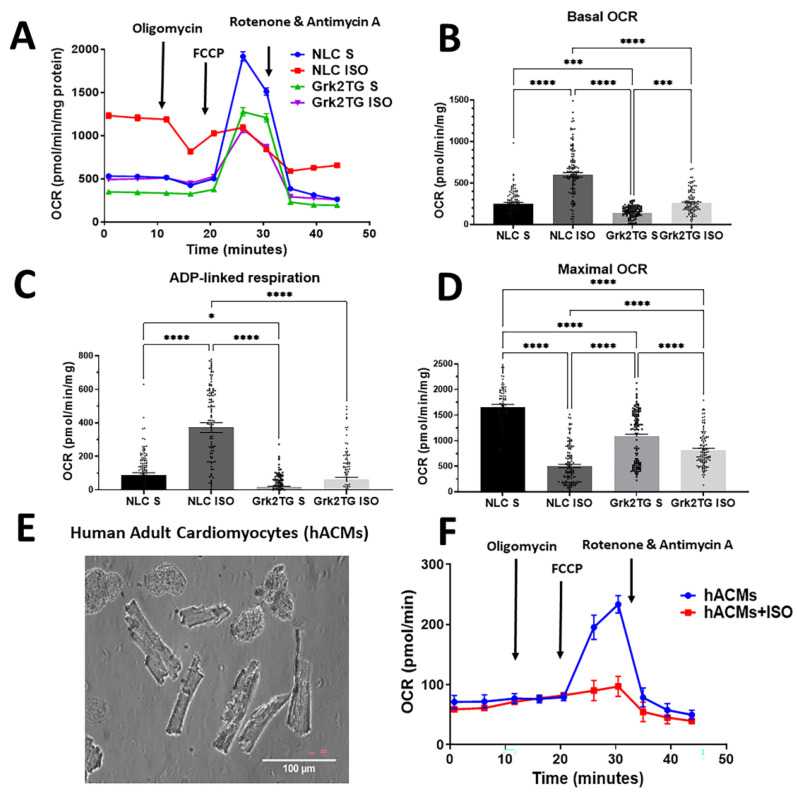
Mitochondrial function responses are altered in response to βAR stimulation and GRK2 expression. (**A**) Overall mitochondrial OCR traces are shown for NLC saline (NLC S; red), NLC ISO (black), Grk2TG + saline (Grk2TG S; blue), and Grk2TG ISO (green) during a mitochondrial stress test (*n* = 114 to 144 wells/condition in 3 independent hearts/condition). (**B**) Quantification of basal respiration. (**C**) ADP-linked respiration, and (**D**) maximal respiration are shown from data in panel A (same *n* value). (**E**) Representative image of hACMs (scale bar is 100 um). (**F**) Mitochondrial OCR in hACMs from the same donor in the presence or absence of ISO, (*n* = 20–25 wells/condition) (mean ± SEM, * *p* < 0.05, *** *p* < 0.005 and **** *p* < 0.0001).

To examine mitochondrial function after chronic ISO stimulation in Grk2TG ACMs, we measured oxygen consumption rates (OCRs) in ACMs from NLC and Grk2TG mice with or without ISO (Figure 4A). Chronic ISO stimulation in NLC ACMs led to a significant increase in basal OCRs and ADP-linked OCRs, with a decrease in maximal respiration and spare capacity (Figure 4A–D). No statistical difference was observed in proton leak between treated and untreated NLC cells. This suggested that NLC ACMs, when exposed to chronic ISO, increased their energetic demand, increasing their coupling efficiency and ATP generation. Contrarily, in unstimulated Grk2TG ACMs, basal OCR was decreased when compared to unstimulated NLC ACMs which was linked to decreased ADP-driven respiration and maximal respiration (Figure 4A–D). Moreover, ISO stimulation in Grk2TG ACMs led to the depression of all respiratory parameters when compared to NLC ISO (Figure 4A–D). This suggests that the upregulation of GRK2 impairs ISO-mediated mitochondrial responses when hearts are chronically stimulated with ISO.

Lastly, we isolated human adult cardiomyocytes (hACMs) (Figure 4E) and assessed acute ISO-mediated mitochondrial respiratory alterations in non-failing hACMs. Human ACMs from the same donor were exposed to either ISO or vehicle for 4 h followed by OCR assessment (Figure 4F). Acute ISO stimulation in hACMs reduced mitochondrial flexibility as ISO decreased maximal respiration. This suggests that acutely ISO depresses maximal respiration prior to changes in baseline respiration. Thus, our data support the notion that ISO alters mitochondrial function and that myocyte GRK2 upregulation negatively impacts ISO-mediated mitochondrial respiration, which we propose contributes to cellular bioenergetic failure.

## 3. Discussion

GRK2 is a kinase known to regulate activated GPCRs and plays an important role in receptor desensitization and recycling [1]. Research spanning over two decades has linked HF to the upregulation of GRK2, subsequent negative cardiac outcomes, and disease progression [1]. We and others have reported that GRK2 is also localized to the mitochondria [5,6,7], where it plays a role in myocardial fatty acid utilization in cardiomyocytes [8,9]. As substrate preference and metabolic pathways are altered during development and cardiac pathologies [10], we sought to further understand how cardiac GRK2 in the adult heart may be altering metabolic networks that facilitate cardiac dysfunction. Our data suggest that indeed the upregulation of GRK2 particularly impairs fatty acid-driven ACM cell survival (Figure 1) which is linked to impaired fatty acid utilization processes known to be involved in the generation of ATP (Figure 2). This is novel in further detailing mechanistically the involvement of this kinase in ACM metabolic regulation. Notably, this is relevant to what is known about ACM metabolite utilization in non-diseased conditions, that is, cellular ATP is mainly derived from fatty acids [13]. Thus, upregulation of cardiac GRK2 in the absence of cardiac injury may pre-dispose the heart for worse outcomes in response to cellular, mechanical, or metabolic stressors.

Clinically, a persistent higher resting heart rate has been reported as an independent risk factor for HF [14], yet this remains a correlative observation. We tested whether extended activation of the βAR system altered mitochondrial responses and whether the upregulation of cardiac GRK2 conformed to the necessary ATP demand elicited by such stimulation. Our data (Figure 3 and Figure 4) support the notion that the upregulation of GRK2 in the mitochondria impairs ISO-mediated mitochondrial respiration and hampers the ability of ACMs to adapt to increased ATP demand elicited by βAR stimulation. This ISO-mediated mitochondrial impact is partially observed in acutely ISO-treated hACMs where a substantial decrease in maximal respiration is noted (Figure 4). Our data in hACMs suggest that short-term βAR stimulation utilizes its maximal respiratory capacity to maintain cellular processes prior to altering baseline respiration. These results converge with work showing that in the absence of cardiac injury, Grk2TG are functionally similar to control mice [1], though ISO stimulation in Grk2TG mice decreases the activity of adenylyl cyclase [12]. We propose that the upregulation of GRK2 in the myocyte limits mitochondrial respiratory responses that link ATP to the generation of cAMP. This in turn could contribute to the observed decrease in adenylyl cyclase activity, ultimately contributing to the decrease in contractile potential and negatively impacting cardiac function.

Our work provides new insight on how GRK2 impacts mitochondrial function as it relates to specific substrate utilization and cell survival mechanisms. In addition, it details GRK2-mediated mitochondrial responses in the presence or absence of adrenergic stimulation. Understanding this early-stage metabolic remodeling can guide novel diagnostic and interventional approaches for HF as well as other diseases such as arrhythmogenic right ventricular cardiomyopathy (ARVC) and catecholaminergic polymorphic ventricular tachycardia (CPVT) where sudden death is often driven by catecholamine overstimulation. Nonetheless, further studies are needed to precisely delineate the link between bioenergetic alterations and the propensity for arrhythmias and sudden death in these patients.

## 4. Materials and Methods

### 4.1. Animal Models

Cardiac-specific GRK2 overexpressing (Grk2TG) [12] and non-littermate control (NLC) adult male mice (10- to 13-week-old) were used. Transgenic GRK2-overexpressing mice were crossed with wild-type C57Bl6 purchased from Jackson Laboratories. Litters were screened for the transgene and pups without the transgene were assigned to the NLC group. All animal experiments were performed with the approval of the Institutional Animal Care and Use Committee at Drexel University.

### 4.2. Mouse Adult Cardiomyocyte Isolation (ACMs)

ACM isolation was performed as published [8].

### 4.3. Substrate-Specific Cell Culture Preparations

ACMs were incubated for 2 h at 37 °C/5% CO_2_ on laminin-coated coverslips. Cells were then cultured at 37 °C in M199 medium (Sigma-Aldrich, St. Louis, MO, USA) with 1% penicillin/streptomycin, 26.2 mM sodium bicarbonate, 25 mM HEPES, containing blebbistatin (Sigma-Aldrich). Substrates were prepared as published [15] (palmitate 50 μM, carnitine 200 μM, glucose 5 mM, pyruvate 100 μM, BCAAs (leucine 500 μM, isoleucine 500 μM, valine 500 μM), and ketone body (β-hydroxybutyrate 0.5 mM) were added and cultured for 24 h.

### 4.4. Live/Dead Assay

The LIVE/DEAD™ Viability/Cytotoxicity Kit (Thermo Fisher Scientific, Waltham, MA, USA) was used per the manufacturer’s directions. At 24 h post isolation, unfixed ACMs were labeled with 2 μM calcein-AM (green) and 2 μM ethidium homodimer-1 (red) for 40 min. Cells were then fixed with 10% formalin solution and mounted with Diamond Antifade Mounting Medium (Thermo Fisher Scientific). Images were acquired using an Olympus BX51 fluorescence microscope and quantified with ImageJ. Ten random fields of view were analyzed per substrate condition. Analysis was performed by taking the ratio of ethidium homodimer-1-positive cells over the total number of platted ACMs/field.

### 4.5. Mini-Osmotic Pump Implantation

Mice were subjected to Alzet miniosmotic pumps containing isoproterenol (30 mg/kg/day, β-adrenergic receptor agonist) or saline for 6–7 days. Pumps were prepared in a sterile environment and primed for at least 2 h at 37 °C. Animals were monitored thereafter for pain or discomfort.

### 4.6. Echocardiography

M-mode echocardiography as published [15].

### 4.7. Mitochondria Respiration Measurement by Seahorse

ACMs were isolated and platted in XF96 Seahorse^®^ plates pre-coated with laminin and incubated with Seahorse Media as published [15] in a non-CO_2_ incubator for 15–20 min. Oxygen consumption rates (OCR) were measured with preloaded drugs including Oligomycin (1.3 μM), FCCP (1 μM), and Rotenone/Antimycin A (1 μM). After the recording was finished, the Seahorse plate was subjected to a protein assay for normalization purposes.

### 4.8. Isotope Tracing Analysis

ACMs from both Grk2TG and NLC were cultured at the same time in M199 medium (Sigma-Aldrich) with 1% penicillin/streptomycin, 26.2 mM sodium bicarbonate, 25 mM HEPES only supplied with (1) 100 μM U-^13^C_16_ Palmitate + 200 μM carnitine or (2) 5 mM U-^13^C_6_-Glucose plus pyruvate (100 μM) for 4 h. ACMs were collected, frozen at −80 °C, and processed then analyzed by liquid chromatography high-resolution mass spectrometry as published [16] to determine the abundance and isotopic patterns of metabolites.

### 4.9. Subfractionation Preparation and Western Blot Analysis

Samples were prepared and mitochondrial and cytosolic fractions were extracted exactly as previously published [15]. Western blotting was conducted to analyze protein levels in specific cellular fractions. Primary antibodies used were: GRK2 (G0296, Millipore Sigma), GAPDH (SC-32233, Santa Cruz), VDAC1 (75-204, NeuroMab). Secondary Antibodies used were: Goat anti-Mouse IgG Alexa Fluor 680 (A-21058, Invitrogen), IRDye^®^ 800CW Goat anti-Rabbit IgG (925-32211, Li-Cor). Imaging was performed using LICOR and densitometry analysis was performed using Image Lab. GRK2 protein levels were normalized against the loading control. NLC S was set to 1 in each experimental gel and used to compare to experimental groups as previously published [15].

### 4.10. Human Adult Cardiomyocytes (hACMs)

Human cardiac tissue was obtained from IRB-approved donations through the Pennsylvania GoL Program. The cardiac donor was a non-failing non-DCD (donor after cardiac death) rejected for transplant. The heart was maintained in transplant solution, never frozen, and directly transported to the laboratory for cellular isolation. Left ventricular tissue was minced in sterile conditions and rinsed in buffer A (mM: NaCl 126, KCl 4.4, MgCl_2_ 5, NaH_2_PO_4_ 5, HEPES 5, Glucose 22, Taurine 20, Creatine 5, pyruvate 5, BDM 10) followed by incubation in buffer A containing collagenase B and protease IX (Millipore Sigma) at 37 °C for 15 min. Three fractions were collected and combined with buffer B (buffer A containing 20% FBS). Fractions were filtered, spun down, and resuspended in buffer B. hACMs were re-introduced to calcium (final concentration of 1.26 mM), and combined for cell counting using a hematocytometer. hACMs were treated with ISO (Sigma-Aldrich) or vehicle for 4 h prior to experiments.

### 4.11. Statistical Analyses

Statistical analyses were conducted using Graph Pad Prism 8.0 software. Data are expressed as the mean ± SEM for the indicated number of experiments or mice. The difference in means between two groups and multiple groups was evaluated using unpaired Student’s *t*-test and one-way ANOVA followed by Bonferroni test, respectively. * *p* < 0.05, ** *p* < 0.01, *** *p* < 0.005 and **** *p* < 0.0001; *p*-value less than 0.05 was considered statistically significant.

## Data Availability

The data that support the findings of this study are available from the corresponding author upon reasonable request.
